# Community-Based Health Insurance Utilization and Its Determinants among Informal Workers: Cross-Sectional Study

**DOI:** 10.4314/ejhs.v33i5.8

**Published:** 2023-09

**Authors:** Tilahun Deresse, Akine Eshete, Hailu Mulatu, Megbar Dessalegn

**Affiliations:** 1 Department of Surgery, School of Medicine, Debre Berhan University, Debre Berhan, Ethiopia; 2 Department of Public Health, Debre Berhan University, Debre Berhan, Ethiopia; 3 Department of Surgery, School of Medicine, Debre Markos University, Debre Markos, Ethiopia

**Keywords:** Berek, Community-Based Health Insurance, Ethiopia, Informal Workers

## Abstract

**Background:**

Ethiopia has implemented a community-based health insurance (CBHI) program to provide coverage to 80% of the population and shield underprivileged individuals from the detrimental effects of exorbitant medical expenses. However, there is a paucity of data regarding its utilization and pertinent concerns. This study aimed to evaluate the utilization of CBHI and its associated factors among informal workers in Berek District.

**Methods:**

This community-based cross-sectional study was conducted between June 15 and July 15, 2022. The sample population comprised 538 households selected using a multistage sampling approach. Data analysis was done using SPSS Version 26. Variables with P-values of less than 0.25 during the bivariate analysis were selected for multivariate analysis using binary logistic regression. The statistical significance threshold was set at a p-value of 0.05.

**Results:**

The utilization of Community-Based Health Insurance (CBHI) was 49.8%. Age between 30 and 39 years, monthly earnings of less than 1500 Ethiopian Birr, presence of chronic illness, membership in social organization, and possessing adequate knowledge were found to have a statistically significant association with the use of CBHI.

**Conclusion:**

The utilization of CBHI was low within the confines of this district Age, income, social group membership, and chronic illnesses were significantly associated with CBHI utilization.

## Introduction

Despite the objectives of strategies for universal health coverage (UHC) to ensure access to a primary care-focused health system and eliminate financial barriers, approximately 150 million individuals encounter financial difficulties annually, and approximately 100 million are pushed into poverty owing to high out-of-pocket (OOP) healthcare expenses (1,2). Financing UHC has proven to be a challenge in numerous low- and middle-income countries (LMICs) as a result of limited financial resources and slow economic growth (3).

More than half of African nations still rely on OOP healthcare, which affects the frequency of use of health services among impoverished rural communities, ultimately affecting the strategy for achieving UHC (4). Community-based health insurance (CBHI) has been established in LMICs to reduce OOP healthcare costs and promote the use of health services to attain the UHC objective (4,5). The CBHI is a voluntary, non-profit insurance that consolidates premium payments from members into a common fund and covers essential medical expenses at nearby clinics when a member falls ill (5).

The CBHI program was introduced in Ethiopia in 2011 with the aim of providing financial assistance to 13 districts in Tigray, Oromia, Amhara, and the Southern Nation Nationalities and People's Region (6-8). Since then, more regions, including Addis Ababa, have been incorporated into the program (9). Ethiopia seeks to achieve UHC by 2035 (4). However, the low utilization of health services (34.3%) compared with other African countries is attributed to inadequate healthcare financing (10). Households with insurance (50.5%) were found to use healthcare services more than those without insurance (29.3%) (7). The success of the CBHI program in improving the health and well-being of different demographic groups remains uncertain and depends on various factors, such as access to medications, chronic illness, recent sickness episodes, and sociodemographic characteristics (11-13).

The achievement of UHC by reducing out-of-pocket (OOP) payments was agreed upon by countries during the WHO General Assembly (14). However, actual spending on healthcare remains below 12%. In low- and middle-income countries (LMICs), OOP healthcare expenditure, which accounts for 40% of overall healthcare spending, has a significant impact on healthcare service utilization (15). The use of direct OOP payments as a healthcare funding mechanism is an unfair and inefficient approach that limits access to healthcare and exposes the population to catastrophic costs (6).

The enrollment rate in the CBHI program in Ethiopia is only 0.2% of the catchment population, a challenge faced by many African nations (16, 17). A study conducted in Southwestern Uganda reported that 25.1% of insurance subscribers dropped out, with household socioeconomic status, higher family size, and distance from the hospital being the primary risk factors (18). While numerous studies have been conducted on CBHI in West Africa and Asia, East Africa has received less attention, particularly regarding the factors that influence CBHI use (16, 19-23)

One of the major public health issues in Ethiopia is the burden of household OOP healthcare expenses, which accounts for 34% of healthcare spending and is among the highest worldwide. This burden is linked to underutilization of health insurance (15). Although the OOP health expenditure in Ethiopia is expected to reduce from 33.7% to 15% by the end of 2020, it currently stands at 31% (24).

Currently, CBHI has expanded to encompass 509 districts. The ultimate objectives of this initiative are to enhance quality, promote financial accessibility, reduce financial burdens (namely, out-of-pocket health spending on households during illness), and mobilize additional resources for the health sector (25). Nevertheless, various investigations have demonstrated that the level of CBHI utilization in Ethiopia falls significantly short of the 80% threshold outlined in Health Sector Transformation Plan II (7, 12, 24, 26, 27). A recent study conducted by the Ethiopian Health Insurance Agency published in September 2020 revealed that the national CBHI utilization rate was 50%. However, it should be noted that the Oromia region displayed the lowest rate of CBHI use among all regions at 44% (28). The ramifications of such low levels of CBHI utilization are substantial, as they not only hinder Ethiopia's progress towards achieving universal health coverage by 2030, but also result in increased out-of-pocket costs and limited health service consumption (10). Even though Ethiopia introduced CBHI in several parts of the country, the pilot study conducted by the Ethiopian health insurance agency (25) has recommended a further investigation into the usage of community-based health insurance. In our study area, the socio-cultural context differs significantly from the pilot district, and our target districts were not included in the pilot study. Moreover, the factors that influence the utilization of CBHI are inadequately described in Ethiopia generally and in our study setting specifically. As a result, the objective of this study is to assess the factors that affect the utilization of community-based health insurance in Berek district.

## Methods

**Study area**: The research was conducted in Berek District, which is one of the districts situated in the Finfine Special Zone in the Central Ethiopia region. The district is found 40 kilometers away from Addis Ababa, the capital city of the nation. The district shares boundaries with the East Shewa Zone of Oromia Region, Addis Ababa city, Sululta Woreda of Finfine Special zone, and North Shewa Zone of Amhara Region (47). The Berek District Health Office provided data indicating that the district has a population of 101,554 individuals residing in 22 rural Kebeles, with 51,793 being male and 49,761 being female. The district has 22 health posts, 4 health centers, and 21,157 households. The district commenced CBHI use in 2016.

**Study design and period**: Community based cross-sectional study multistage sampling technique was conducted from June 2022 to July 2022. All households in the district were taken as the source populations, and the study populations were households in the selected Kebeles. Sampled household heads/spouses with informal work and lived for at least 6 months in the selected Kebeles of Berek district were included in the study. All household heads/spouses with critical illness were excluded.

**Sample size determination**: The sample size for CBHI coverage was calculated by using the single population proportion formula, n = [(Zα/2)^2^ x P x (1-P)] /d^2^ ], considering 95% level of confidence, 80% power and proportion of 66.3% from previous similar study conducted in Ethiopia (12), making the sample size to be 343. By considering 10% non-response rate and 1.5 design effect [(343+34) x 1.5], the final sample size was 566.

**Sampling technique and procedures**: Berek district has 22 Kebeles, of which 7(32%) were included in the study, as observed in a previous study (8). These Kebeles were randomly selected using a lottery method. Subsequently, a sampling frame consisting of lists of households was obtained from the family folder available for each kebele. Respondents were selected from the frame lists of households using systematic random sampling methods across the seven kebeles. Finally, the calculated sample size was distributed to each selected kebele to obtain the final study participants (households), using the formula ni = n (Ni/N), where ni represents the sample in each kebele, n represents the sample size (566), Ni represents each kebele's household, and N represents the total number of households (6440) of the seven Kebeles. The interval (k) was calculated by dividing the total number of households found in the seven Kebeles by the total sample size (6440 households divided by 566), resulting in 11. Subsequently, the study households in the seven Kebeles were selected by utilizing the systematic random sampling technique, beginning from the indexed household and every 11th interval until the required sample size was reached.

**Data Collection Methods and Tools**: Data were collected by seven diploma nurses from all the selected households using a pre-tested questionnaire.

**Data Quality Control**: Individuals tasked with collecting and overseeing data were provided with comprehensive training for a day on the study's objectives, effective interviewing techniques, proper questionnaire administration, and identification of skipping patterns. Prior to actual data collection, a pretest was conducted on a 5% sample size in the Jidda district to assess the questionnaire's clarity and sequence of questions. Based on the results, questions that were deemed unclear or confusing were refined. To ensure completeness, the supervisor checked the data daily during the data-collection period. Additionally, each questionnaire was assigned a unique identification number as a variable for data entry on the computer. Epidata software was employed to ensure completeness and facilitate the easy tracking of potential data entry errors. Furthermore, multivariable analysis was conducted to control for confounding variables.

**Dependent variable**: Utilization of community based health insurance utilization

**Independent variables**: Socio-demographic factors: Age, sex, family size, education, occupation, marital status and income.

**CBHI related factors**: Affordability of premium, trustworthiness of management, knowledge of scheme, membership in social association, exposure to source of credit, source of information about CBHI and time interval of premium payment

**Health related factors**: Distance of health facility, perceived quality of health care, presence of chronic illness in households, traveling time to nearest health facility, waiting time for health service and presence of frequent ill household member

### Operational definitions

**Informal workers**: Households who are living on agriculture, trade and private micro businesses (6).

**Good knowledge**: Respondents who scored mean and above on 6 CBHI knowledge assessing questions. Otherwise, respondents were considered as having poor knowledge (5).

**Presence of Chronic disease in the household member**: When one of the household family members had disease for more than one month (6)

**Perceived quality of health care**: Extent of the community's views on the quality of health service delivery and is measured on a five item Likert scale from very poor to very good (37)

**Trustworthiness**: measured by five item Likert scale and finally merged in to two to assess the characteristic (6)

**Data processing and analysis**: The collected data were validated, encoded, and inputted into Epidata Version 4.6. Subsequently, the data were exported to the Statistical Package for Social Sciences (SPSS) version 26.0. The results are presented using texts, tables, and figures, utilizing descriptive statistics. Furthermore, binary logistic regression analysis was conducted to examine the independent and outcome variables. To determine the components linked to the use of CBHI, a bivariate study was conducted, wherein the proportion and crude odds ratio (COR) of each independent variable against the outcome variable were calculated.

To account for potential confounders and identify significant factors related to an outcome variable, independent variables with a P-value of 0.25 in bivariable logistic analysis were included in the final multivariable logistic regression model. Multicollinearity was assessed using the Variance Inflation Factor (VIF), where variables with a VIF <10 were included in the bivariable analysis and variables with a VIF > 10 were merged prior to analysis. The Hosmer–Lemeshow test was employed to assess the goodness of fit of the model to the data. As the test yielded a significant value of 0.431 and the observed and predicted models were congruent, the model was deemed adequate. Finally, the adjusted odds ratio (AOR) with 95% confidence interval (CI) was calculated to determine the strength of this relationship. In the multivariable analysis, a P-value of 0.05 was considered statistically significant.

**Ethical considerations**: The Asrat Waldeyes Health Science Campus Institutional Review Board at Debre Berhan University approved this study. Seven Kebeles were selected, and an official consent letter was obtained from the district health office. The confidentiality of participants was ensured by excluding their names from the collected data. Informed consent was obtained from each participant after providing a comprehensive explanation of the study objectives, duration, potential risks, and benefits. All the respondents provided written consent.

## Results

**Socio-demographic characteristics**: The study included 538 households with a response rate of 95%. The participants' ages ranged from 18 to 76 years, with an average age of 43.30 ± 12.87 years. Among the participants, 445 (82.7%) were married, and 485 (90.1%) engaged in agricultural pursuits. The male gender constituted 92% of the participants, with 219 (40.7%) being literate. The household population averaged at 5.41 with a standard deviation of 2.46, and the range was from 1 to 13 members. In addition, 302 (56%) participants had a monthly income of less than 3000 Ethiopian Birrs (ETBs) (Table 1).

**Table 1:** Socio-demographic characteristic of the study participants on community-based health insurance utilization and associated factors in Berek district, 2022 (n =538)

Variables	Frequency (%)
Age	
<30	95 (17.7)
30-39	121 (22.5)
40-49	153 (28.4)
50-59	97 (18)
≥60	72 (13.4)
Gender	
Male	499 (92.8)
Female	39 (7.2)
Ethnicity	
Oromo	522 (97)
Amhara	16 (3)
Religion	
Orthodox Tewahido	478 (88.8)
Muslim	36 (6.7)
Protestant	24 (4.5)
Marital status	
Married	445 (82.7)
Widowed	24 (4.5)
Divorced	42 (7.8)
Separated	1 (0.2)
Single	26 (4.8)
Educational status	
Unable to read and write	150 (27.9)
Able to read and write	219 (40.7)
Primary education	35 (6.5)
Secondary education	130 (24.2)
College and above	4 (0.7)
Occupational status	
Farmer	485 (90.1)
House wife	32 (5.9)
Merchant	21 (3.9)
Family size	
<5	256 (47.9)
≥5	282 (52.4)
Monthly income (ETBs)	
<3000	302 (56.1)
3000-5000	100 (18.6)
>5000	(25.3)

**Community-based health insurance utilization**: In this study, 298 of the 538 participants (55.4 %) were identified as members of the CBHI program. Of these, 268 (89.9%) had renewed their membership cards. However, 30 families (5.6%) who had utilized the service in the previous two–three years did not renew their membership cards. The CBHI utilization rate was 49.8% (268). Notably, 59.5 majority of the study participants (59.5%) had acquired knowledge about community-based health insurance from medical experts. In addition, 394 homes (73.2%) were members of social associations, whereas 320 households (81.2%) participated in Iddir (Table 2).

**Table 2:** Community based health insurance related responses of study participants on CBHI utilization and associated factors among informal workers in Berek district, 2022

Variables	Categories	Frequency (%)
From you heard about the CBHI scheme?	Health professionals	320 (59.5)
	Mass media: ETV, radio	108 (20.1)
	Health professionals & Mass media	106 (19.7)
	CBHI officials in public meeting	4 (0.7)
Are you a member of CBHI?	Yes	298 (55.4)
	No	240 (44.6)
Have you renewed your membership card? (N=298)	Yes	268 (89.9)
	No	30 (10.1)
Have you ever been a member of a social association?	Yes	394 (73.2)
	No	144(26.8)
Which social association you are /were member? (N=394)	Debo	6 (1.5)
	Equb	63 (16)
	Iddir/kire	320 (81.2)
	Credit & saving	5 (1.3)
Does your household currently have outstanding loans/credit?	Yes	108(20.1)
	No	430 (79.9)
Source of loan/credit (N=108)	Microfinance	32(29.6)
	Money Lender	44(40.8)
	Friends/Neighbors	32(29.6)

**Knowledge of study participants about CBHI**: Among the entirety of the households that were encompassed within the scope of this particular study, it was revealed that 256 (47.6%) of them, possessed a commendable level of knowledge with regards to the matter of CBHI, as evidenced by the data provided in Table 3.

**Table 3:** Knowledge questions and responses on community-based health insurance utilization and associated factors among informal workers in Berek District, 2022

Knowledge Questions	Responses		

	Correct	Not correct	Do not know

	Freq (%)	Freq (%)	Freq (%)
Only people who fall sick should consider buying CBHI?	251 (46.7)	**273(50.7)**	14 (2.6)
In CBHI program you have to pay money but don't know whether you will get money back?	134 (24.9)	**367 (68.2)**	37 (6.9)
CBHI program is like savings scheme; you will receive interest and get your money back?	70 (13)	**366 (68)**	102 (19)
In CBHI program you pay money for the CBHI to finance your future health care needs	**151 (28.1)**	278 (51.7)	109 (20.3)
All health care costs will be covered by CBHI program	153 (28.4)	**275 (51.1)**	110 (20.4)
If you do not make claim any costs through CBHI your premium will be returned	47 (8.7)	**(5.6)**	138 (25.7)

**Reasons for CBHI utilization**: The primary determinant for membership in the CBHI program was identified as frequent illness among household members (52.3%) and to finance healthcare expenses (34.6%). Others joined the program for reasons, since exempted from registration and premium fees (9.1%), and because they thought children need health services (4%).

**Reasons for dropping out or not being member in CBHI**: Factors mentioned by the households for drop outs after being enrolled in CBHI were, infrequent illness or injury (16.6%), expensive registration and premium fees (6.7%), low-quality health services (26.7%), and an unsatisfactory benefit package (50%). It is noteworthy that 46.7% of the households that dropped out initially intended to renew their membership cards. Furthermore, in this district, 50.2% of the households (270) did not opt for the CBHI program. The primary reason cited for non-enrollment was the lack of regular occurrences of illnesses and injuries within the household (51.3 %). Lack of knowledge about CBHI (17.1%), and shortage of money (17.1%) were other reasons raised by the households.

**Health and Health Related Characteristics of Study Participants**: Based on the data presented in Table 4, a significant proportion of individuals, 461 (85.7%) opted to seek medical attention from a public health center when they fell ill. Furthermore, 305 (56.7%) participants chose the aforementioned facility because of the provision of high-quality medical care. The research also revealed that 101 (18.8%) families had at least one member afflicted with chronic diseases. Additionally, 166 households (30.9%) had at least one individual who had been unwell within the three months preceding the data collection. Notably, all of these individuals received treatment. Pertaining to proximity to medical institutions, the data suggest that the average distance from the respondents' homes to the nearest medical facility was 8.93 km, with a minimum distance of 1 km and a maximum distance of 21 km. Furthermore, the study found that the average travel time to the nearest medical center was 88 minutes, with travel times varying between 15 and 210 minutes.

**Table 4:** Health and health related characteristics of respondents among informal workers in Berek district, 2022

Variables	Category	Frequency (%)
Where do you get treatment when you sick	Private Heath Facility	25(4.6)
	Public health center	461(85.7)
	Public hospital	52(9.7)
Reasons for going to health facility	Physically accessible health facility	192(35.7)
	Cheaper health service cost	23(4.3)
	Short waiting time	18(3.3)
	Effective health service	305(56.7)
Presence of chronic disease?	Yes	101(18.8)
	No	437(81.2)
Any illness encountered in the past 3 months	Yes	166(30.9)
	No	372(69.1)
Health care cost coverage (N=166)	Self	38(22.9)
	Free	128(77.1)
Perceived quality of the health care service given	Very low	1(0.6)
	Neutral	14(8.4)
	High	48(28.9)
	Very high	103(62)

**Factors associated with community-based health insurance utilization**: Various sociodemographic and economic factors were found to have a significant relationship with CBHI program utilization in the bivariate logistic regression test. These factors included age, marital status, family size, level of education, monthly income, awareness of CBHI, length of time between premium payments, affordability of registration fees and premium payments, management official trustworthiness, membership in social associations, exposure to sources of credit/loans, presence of chronic illness at home, proximity to health facilities, and travel time to the closest health facility (p<0.25).

In the adjusted model, it was observed that individuals who were household heads between the ages of 30-39 years (AOR:3.08, 95%CI: (1.23, 7.82)), 40-49 years (AOR:2.63, 95%CI: (1.09, 6.35)), and 50-59 years (AOR:3.13, 95%CI: (1.16, 8.43)), whose average monthly income was less than 1500 ETB (AOR:7.58, 95%CI: (4.21, 13.63)), and who agreed with the trustworthiness of CBHI officials (AOR:4.15, 95%CI: (1.26, 13.62)), as well as those who had a chronic disease in their family members (AOR:3.82, 95%CI: (1.53, 9.53)), were members of social associations (AOR:3.34, 95%CI:(1.60,6.97)), and had good knowledge about CBHI utilization (AOR:2.82, 95% CI:(1.43,5.56)), were more likely to utilize CBHI services (Table 5).

**Table 5:** Factors associated with community-based health insurance utilization among informal workers in Berek district, Multivariable analysis, 2022 (n=538)

Variables	Category	CBHI utilization		COR (95% CI)	AOR (95%CI)

		No (%)	Yes (%)		
Age	<30	62(65.3)	33(34.7)	1	1
	30-39	55(45.5)	66(54.5)	2.26(1.30, 3.922)	3.08(1.22,7.82)*
	40-49	70(45.8)	83(54.2)	2.23(1.31, 3.780)	2.63(1.09,6.35)*
	50-59	52(53.6)	45(46.4)	1.63(0.91, 2.91)	3.13(1.16,8.43)*
	>=60	31(43.1)	41(56.9)	2.49(1.32, 4.66)	2.24(0.81,6.20)
Marital status	Married	213(47.9)	232(52.1)	1.73(1.09, 2.72)	0.56(.27.1.13)
	Other*	57(61.3)	36(38.7)	1	1
Family size	<=5	207(64.5)	114(35.5)	1	1
	>5	63(29)	154(71)	4.44 (3.06, 6.44)	0.75(0.40,1.43)
Educational status	No formal	90(60)	60(40)	1	1
	Formal	180(46.4)	208(53.6)	1.73 (1.18, 2.54)	1.15(0.62,2.14)
Monthly income	<=1500	31(15.9)	164(84.1)	12.16 (7.77, 19.02)	7.58(4.21,13.63)**
	>1500	239(69.7)	104(30.3)	1	1
Time interval for premium payment	Disagree	239(70.9)	98(29.1)	1	1
	Agree	31(15.4)	170(84.6)	13.37(8.53,20.96)	1.84(0.57,5.95)
Registration fee affordability	Disagree	234(71.3)	94(28.7)	1	1
	Agree	36(17.1)	174982.9)	12.03(7.82,18.52)	0.61(0.17,2.17)
Social association	Yes	152(38.6)	242(61.4)	7.226(4.51,11.57)	3.34(1.60,6.97)**
	No	118(81.9)	26(18.1)	1	1
Premium payment affordability	Disagree	241(71.3)	97(28.7)	1	1
	Agree	29914.5)	171(85.5)	14.65(9.26,23.18)	1.631(0.45,5.93)
Trust on CBHI official	Disagree	244(72.6)	92(27.4)	1	1
	Agree	26(12.9)	176(87.1)	17.95(11.15,28.91)	4.15(1.26,13.62)*
Outstanding loan/credit	Yes	39(36.1)	69(63.9)	2.05(1.33,3.18)	1.85(0.95,3.62)
	No	231(53.7)	199(46.3)	1	1
Chronic disease	Yes	10(9.9)	91(90.1)	13.37(6.77,26.39)	3.82(1.53,9.53)*
	No	260(59.5)	177(40.5)	1	1
Traveling time to health facility	< 60 Min.	74(36.5)	129(63.5)	3.14(1.01, 9.71)	0.55(0.07,4.29)
	60-120 Min	124(54.9)	102(45.1)	1.48(0.48, 4.56)	0.37(0.06,2.38)
	121-180Min	63(66.3)	32(33.7)	0.91(.283, 2.96)	0.27(0.06,1.29)
	> 180 Min.	9(64.3)	5(35.7)	1	1
Distance of health facility	<=5 Km	56(37.1)	95(62.9)	6.79(3.40, 13.55)	0.99(0.17,5.82)
	>5-10 Km	108(51.4)	102(48.6)	3.78(1.94, 7.3)5	0.84(0.18,3.81)
	>10-15 km	54(48.2)	58(51.8)	4.3(2.11, 8.76)	1.39(0.39,4.93)
	>15 km	52(80)	13(20)	1	1
Knowledge on CBHI	Poor	217(77)	65(23)	1	1
	Good	53(20.7)	203(79.3)	12.79(8.49,19.27)	2.82(1.43,5.56)*

Notes: Other*= House wife and merchant, 1= Reference

* = P value < 0.05 (significant)

** = P value ≤ 0.001(highly significant)

## Discussion

This study discovered that the prevalence of utilizing the CBHI program in the district was found 49.8% [95% CI: 45.7, 54.3)]. This outcome was similar to that reported in Uganda (48.3%) (31). One possible explanation for this similarity is that the CBHI utilization strategies are analogous in both countries. However, this finding was inferior to the results of a study conducted in Pakistan (64%) (29), the Amhara region of Werebabu district (73.6%), Simada district (89%), West Gojjam (57.8%), East Gojjam (81.5%), Bugna district (77.8%), and Dessie town (82.3%) (4, 8, 32–35). Furthermore, it was also lower than that in studies conducted in the Akaki district (66.3%) and among urban dwellers in the Oromia region (86.3%) (12, 38). Nevertheless, the level of CBHI utilization in Cameroon (2.4%) (21), Nigeria (4.5%) (30), Southern Ethiopia (33.3%) (26), Sidama Region in 2019 (12.8%) (36), 2020 (20.2%) (37), and the Oromia region of Gida Ayana district (27.5%) (6) was less than that in Berek district at the time. These variations in CBHI utilization can be attributed to temporal and geographic differences, societal values, lifestyles, socioeconomic conditions, and the dissemination of health information. This highlights the fact that owing to constrained access to health education regarding community-based health insurance, the level of awareness created for inhabitants differs from nation to nation.

According to the results of a multivariable logistic regression analysis, a strong correlation exists between the age of household heads and their utilization of CBHI. Specifically, individuals in the 30- to 39-year-old age range, who may be considered relatively young, are approximately three times more likely to utilize CBHI. This finding is consistent with that of previous research conducted in Nepal (39), which indicated that older individuals were less likely to enroll in the CBHI program. However, this result contrasts with research conducted in Cameron (21), Senegal (23), and Ethiopia's Gida Ayana and Manna districts, where respondents were more likely to use CBHI as they aged. This discrepancy may be attributed to the fact that younger individuals tend to be more productive and have the financial means to afford premium payments, thereby ensuring a higher level of healthcare consumption at a lower cost. This study found a statistically significant connection between the average monthly income of households and CBHI utilization. Households with a monthly income of less than 1500 ETB were found to be 7.6 times more likely to use community-based health insurance than those with monthly incomes of more than 1500 ETB. Conversely, the findings from other studies do not align with this result. Studies from Pakistan (29), Cameroon (21), and Uganda (22) revealed that higher-income households are more likely to use the CBHI. However, comparable studies conducted in Simada District, East Gojjam, Dessie Town, and Bugna District have found that individuals were more likely to use CBHI if they belonged to a medium or wealthy family (8, 33–35). The current high cost of healthcare services, which poses a challenge for low-income households to pay out-of-pocket, could be the reason for this discrepancy. This study found a positive and statistically significant relationship between CBHI consumption and management reliability. Those who agreed with management officials' dependability increased their likelihood of using CBHI by 4.1 times. Other studies conducted in Uganda (46), Gida Ayana district (6), Segen region and South Omo zones (26), and Tehulederie and Kalu (43) found a significant positive connection between trustworthiness and CBHI use, corroborating this conclusion. This may be because reliability makes it enjoyable for households to pay their premiums.

In this study, there was a statistically significant correlation between belonging to a social organization, such as Iddir, and community-based health insurance; households with members of social organizations were approximately 3.3 times more likely to use community-based health insurance than households without members. This result conflicts with that of a study conducted in the Werebabu region, which found that households that belonged to a social association (Idir) were 54% less likely to enroll in a CBHI program than those that did not (35). This contentious issue could be the result of differences in the study location and the sociodemographic characteristics of the participants.

The investigation's supplementary discovery revealed a noteworthy correlation between CBHI utilization and the incidence of chronic illnesses among household members. Families with an individual who had a chronic condition were 3.8 times more inclined to use CBHI as opposed to families who did not have such a member. This finding aligns with those of studies conducted in Nepal (39), where households with chronic illnesses in their kin were more likely to participate in CBHI. Results from studies conducted in the Simada district (8), Segen area and South Omo zone districts (26), Southern Ethiopia's Bugna district (34), and Tehulederie, Kalu, and Werebabu districts (43) were also congruent with the current research findings. A possible rationale behind this phenomenon is that households with chronic illness sufferers may require frequent medical services, and CBHI mitigates financial risks for households while also increasing their reliance on the program, particularly for those with lower monthly earnings. CBHI utilization is positively influenced by an individual's knowledge of the program. In one study, participants who possessed knowledge about community-based health insurance were 2.8 times more likely to utilize health insurance than those who did not possess the same knowledge. This conclusion is supported by a study conducted in Nepal that discovered that individuals who were aware of the program were 28.97 times more likely to enroll in health insurance than those who were not aware of it (39). The idea that knowledge is positively associated with the utilization of community-based health insurance is further reinforced by studies conducted in Nigeria (30), Uganda (42, 45, 46), Werebabu district (4), Gida Ayana district (6), and districts in the Segen region and South Omo zones (26). These studies suggest that information can alter people's health-seeking behaviors and enhance their comprehension of the benefits and drawbacks of health-service programs, leading to an increase in the consumption of community-based health insurance.

In conclusion, the utilization of CBHI was observed to be at a low level. The variables that had a significant impact on the utilization of CBHI encompassed the age of the household heads, monthly income, presence of chronic ailments, and membership in social organizations. In addition, knowledge of CBHI, as well as the dependability of CBHI management authorities, was also found to be a crucial determinant of its use.

Based on the findings of this study, the authors provide subsequent recommendations. The regional health bureau ought to concentrate on accomplishing the national aim of CBHI utilization by extending the CBHI management system to the Keble level. To promote utilization, the district health office should establish CBHI education programs directed towards enhancing communities' comprehension of CBHI principles. It is advised that members of the CBHI attend CBHI-related meetings and urge households with higher incomes; no members who are chronically ill, who are not members of social organizations, and who are older to enroll in the program. Officials in charge of district health insurance should take measures to enhance the dependability of CBHI management systems. To acquire a better understanding of the factors influencing the use of community-based health insurance, researchers should concentrate on aspects linked to CBHI utilization and engage in more qualitative research.

## Figures and Tables

**Figure 1:**
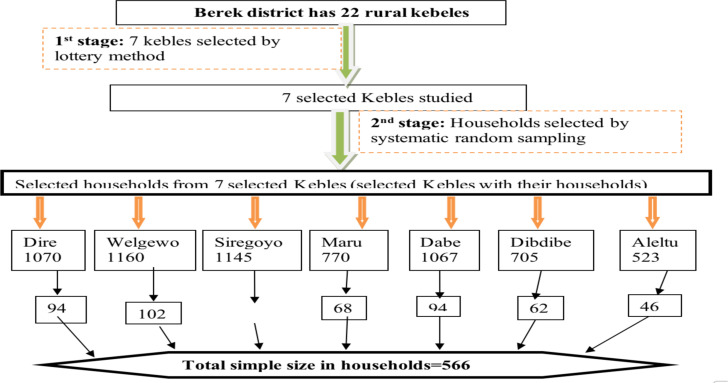
Schematic presentation of the sampling method to assess community-based health insurance utilization and its associated factors in Berek district, 2022
